# Humanized Care in Nursing Practice: A Phenomenological Study of Professional Experiences in a Public Hospital

**DOI:** 10.3390/ijerph22081223

**Published:** 2025-08-06

**Authors:** Monica Elisa Meneses-La-Riva, Víctor Hugo Fernández-Bedoya, Josefina Amanda Suyo-Vega, Hitler Giovanni Ocupa-Cabrera, Susana Edita Paredes-Díaz

**Affiliations:** Grupo de Innovación Humanizadora, Universidad César Vallejo, Av. Alfredo Mendiola, Lima 15311, Peru; jsuyov1@ucv.edu.pe (J.A.S.-V.); hocupa@ucvvirtual.edu.pe (H.G.O.-C.); sparedes@ucv.edu.pe (S.E.P.-D.)

**Keywords:** humanized care, nurses, organizational policy, professional identity, emotional intelligence, competencies, patient-centered care

## Abstract

This study aims to understand the meaning nursing professionals attribute to their lived experiences of providing humanized care within a public hospital setting. Grounded in Jean Watson’s theory of human caring, the research adopts a qualitative, descriptive phenomenological design to capture the perceptions and emotions of nurses regarding humanized care. Data were collected through semi-structured interviews with nine experienced nurses, selected through purposive sampling. The interviews, conducted virtually between July and December 2024, were analyzed using Colaizzi’s method and supported by Atlas.ti software. Four main thematic categories emerged: institutional health policies, professional image and identity, strengths and challenges in care, and essential competencies for humanized care. The findings highlight the critical role of empathy, cultural sensitivity, ethical commitment, and emotional presence in delivering compassionate care. Participants emphasized that, beyond clinical procedures, humanized care requires relational and contextual sensitivity, often hindered by institutional limitations and excessive administrative burdens. The study concludes that nursing professionals are key agents in promoting ethical, empathetic, and culturally respectful practices that humanize health services. These insights offer valuable contributions for designing policies and training strategies aimed at strengthening humanized care as a cornerstone of quality healthcare systems.

## 1. Introduction

In the past decade, the effective exercise of the right to health has faced significant structural barriers and fragmented hospital care, impacting both users and healthcare professionals. This reality was further exposed by the pandemic, which revealed the overload, emotional exhaustion, and resource shortages experienced by healthcare professionals—especially nursing staff, whose work is fundamental to ensuring comprehensive, ethical, and humanized care [1,2]. In response to this situation, organizations such as the World Health Organization [3] have promoted Universal Health Coverage (UHC) globally, with a focus on equity and quality of care, recognizing that millions of deaths each year are attributable to poor quality care or lack of access to health services.

The persistence of traditional biomedical models and hospital management with limited patient orientation has heightened the urgency to redesign care approaches by integrating essential values such as empathy, compassion, respect for cultural diversity, and the promotion of self-care. In this context, nursing emerges as a key actor in leading the transformation toward care environments that dignify human life in all its stages. Drawing on the theoretical contributions of Jean Watson and other contemporary perspectives [4,5], humanized care is emphasized as a relational, holistic, and sensitive practice that acknowledges suffering and responds with authentic presence.

It is essential to recognize that experiences related to humanized care—both positive and negative [6,7,8]—demand deep reflection on care practices. These experiences reveal the urgent need to rethink and redesign care approaches, especially in light of the complexity and challenges of current healthcare contexts. Humanizing care is not merely an ethical aspiration, but a concrete responsibility that requires sensitivity, commitment, and ongoing adaptation to the realities of patients and their families [6].

Humanized care goes beyond the mere application of clinical procedures; it requires that every action be imbued with deeply human qualities such as active listening, compassion, solidarity, empathy, and motivation. Added to this is the imperative to recognize and integrate cultural care, respecting the diversity and multiculturalism of the people being served [9,10,11]. In the delivery of health services, seemingly simple yet powerful gestures—such as a word of encouragement, a look of understanding, or an attitude of hope—can have a profound impact, inspiring trust and strengthening the therapeutic bond, thus facilitating the active participation of the patient and their family in their own care process. Likewise, humanization involves promoting health education from a preventive and formative perspective, encouraging individual responsibility and a commitment to self-care as essential pillars of well-being [12,13,14].

From a theoretical perspective, Jean Watson posits that the role of nursing should focus on providing care that not only meets basic needs but also promotes comfort, alleviates suffering, and contributes to the physical, psychosocial, and spiritual well-being of the human being [2,15]. This approach also involves adopting behaviors and practices that minimize the risks and costs associated with healthcare, without undermining the quality and dignity of care [16,17,18,19]. Humanized care, in this sense, is conceived as an integral, unique, and deeply relational act, grounded in a transpersonal and interpersonal bond between the nurse and the patient—an intersubjective connection where emotions, meanings, and experiences are shared, creating a space of trust, respect, and authentic presence [6,9,20].

Humanized care aimed at patients seeks the highest level of well-being, even during the final stages of life [21], requiring a sensitive and loving approach capable of addressing the invisible aspects of pain and the fear of death [22,23]. It is crucial to integrate technology to monitor and promote humanizing actions that reflect the compassionate dimension of a trust-based environment [24,25], within a framework where health policies are effective and efficient [26,27]. The integration of technology to support humanized care refers to digital tools (such as patient feedback apps, electronic health records with empathy metrics, and telehealth platforms) that enhance nurse-patient communication and monitor compassionate practices. These tools help capture emotional and relational aspects of care in real time, supporting reflective practice and institutional learning [28,29].

The management of nursing care in professional practice must strengthen values such as solidarity, respect, and empathy among teams, honoring individual cultures and beliefs [30]. Providing quality care not only involves improving indicators but also ensuring that the care experience becomes a form of learning that empowers the patient for self-care, thereby contributing to the reduction in avoidable mortality, human suffering, and significant economic losses. This study seeks to reveal the essence and significance of humanized healthcare as experienced by nursing professionals, focusing specifically on the emotional, relational, and ethical dimensions of their lived experiences, rather than describing procedural aspects.

## 2. Materials and Methods

### 2.1. Design

The research was conducted using a qualitative approach, with a descriptive phenomenological design, employing individual semi-structured interviews as the data collection technique. These types of studies begin with a preliminary approach to reality, offering an innovative alternative for the generation of new scientific knowledge [31]. Furthermore, they delve into subjectivity through the lenses of phenomenology and hermeneutics, enabling the extraction of relevant information from existing reality [32,33].

This design was chosen because it allows the exploration of the lived experiences of nursing professionals regarding humanized care, capturing their perceptions, emotions, and meanings associated with this phenomenon. The descriptive phenomenological approach is particularly suitable when the objective is to understand how patients make sense of specific experiences in their professional context.

This qualitative study was reported in accordance with the Consolidated Criteria for Reporting Qualitative Research (COREQ) 32-item checklist, which provides a structured framework to ensure transparency and methodological rigor in qualitative research design, data collection, analysis, and reporting [34].

This study explicitly employed Colaizzi’s descriptive phenomenological method [35], which is particularly appropriate for exploring the lived experiences and the meanings individuals assign to specific phenomena. Colaizzi’s method was implemented as follows: (1) thoroughly reading and rereading all detailed field notes; (2) extracting significant statements directly related to the phenomenon of humanized care; (3) deriving meanings from these significant statements; (4) organizing formulated meanings into thematic clusters; (5) synthesizing these themes into an exhaustive description of the phenomenon; and (6) validating findings through member checking with participants to confirm that the descriptions accurately reflected their experiences. This rigorous methodological process ensured that the phenomenological analysis authentically captured the lived experiences of nursing professionals.

### 2.2. Reflexivity and Researcher Characteristics

The lead interviewer (M.E.M.L.-R.) is a female nurse with a Master’s degree in Nursing Care Management, a Master’s degree in University Teaching, and a Ph.D. in Public Management and Governance. At the time of the study, she was serving as a full-time professor and researcher at Universidad César Vallejo. All interviews were conducted by her, leveraging her extensive experience in qualitative research, nursing care, and public service evaluation. No prior relationship existed between the interviewer and the participants. Participants were informed of the interviewer’s academic background and her interest in advancing humanized care practices in the healthcare sector. To maintain rigor and transparency, the researcher kept a reflexive journal throughout the study to document assumptions, emotional responses, and methodological decisions. In addition, peer debriefing sessions were conducted with co-researchers to ensure credibility and minimize bias.

### 2.3. Participants

Participants were selected using purposive sampling to ensure the inclusion of individuals with rich and diverse experiences related to the phenomenon of interest. The research team contacted potential participants through professional networks and institutional referrals, ensuring that all participants were affiliated with a single public hospital, where the study was exclusively conducted.

A total of nine (9) key participants were included in the study—nursing professionals with an average age of 45. The inclusion criteria required participants to hold at least a bachelor’s degree in nursing. To ensure alignment with the study’s objectives, only nurses actively engaged in direct patient care, clinical supervision, nursing education, or service management were eligible. All participants were employed in a public hospital and held professional roles such as clinical bedside nurse, nurse supervisor, department head, or nurse educator. This role-based selection ensured that participants possessed relevant, diverse, and firsthand experience with humanized care practices within institutional settings. Additionally, participants were required to be at least 25 years old and have a minimum of five years of professional experience. This experience threshold resulted in the exclusion of several potential candidates who did not meet the minimum criteria. This threshold was established to ensure participants had substantial professional exposure, allowing them to reflect on a diverse range of care experiences.

Twelve nurses were invited to participate. Three declined due to scheduling conflicts or lack of availability, resulting in a final sample of nine participants who met the eligibility criteria.

### 2.4. Data Collection

Between July and December 2024, nine interviews were conducted with licensed nurses, holding specializations and assuming roles such as clinical-educational nurse, service area head, supervisor, researcher, head of the nursing department, or nursing director. This information was verified by the research team through the official website of the Ministry of Health (MINSA) and private institutions.

The interviews were conducted via video calls using WhatsApp, each lasting approximately 30 min. Due to the challenge of coordinating a single time slot because of the participants’ varied work schedules, this method was chosen to facilitate participation. At the explicit request of most participants, the interviews were not recorded. Invitations to participate were sent one month in advance via WhatsApp. The interview guide was designed by the researcher and evaluated by five experts who made recommendations to ensure the inclusion of key points relevant to the topic. Interviews were conducted until theoretical saturation was achieved [36].

The structure and formulation of the interview guide were informed by qualitative research practices for phenomenological designs, particularly emphasizing open-ended, experiential questions [37,38], enabling participants to articulate their lived experiences in depth. The specific item numbers and questions included in the guide are presented in Table 1.

The interviews enabled an in-depth understanding of the thoughts, emotions, and actions of the individuals involved in the study. The use of these instruments generated a significant richness of data [39], as the qualitative approach allows for the formulation of questions before, during, and after data collection and analysis [39], recognizing the interdependence between the researcher and the research process [32]. The experiences gathered offer a comprehensive perspective that fosters critical reflection on the phenomena studied [40,41].

In qualitative research, the researcher’s perspective and involvement can influence the data collection and analysis process. In this study, the primary researcher maintained a reflective journal throughout the research to document personal biases, emotional responses, and decisions made during the study [42]. This reflexive approach aimed to enhance the transparency and credibility of the findings. Regular peer debriefing sessions were also conducted to discuss potential biases and interpretations with fellow researchers.

It is important to highlight that humanized care significantly contributes to health services, providing sensitive and compassionate attention that offers a sense of security amidst the uncertainty caused by illness. However, these competencies are often lost over time due to the assumption of unrelated and distracting responsibilities that impact the professional’s work [43].

The interviews were collected under the title “Building Humanized Care.” For qualitative data analysis, Atlas Ti software was used to construct the pre-established codes and subcategories: (a) Institutional health policies, (b) Professional image and identity, (c) Strengths and challenges in care, and (d) Competencies for humanized care. Based on the results, keywords and recurring expressions were identified within the coded subcategories, allowing for the creation of conceptual networks represented in the figures presented.

Data analysis followed Colaizzi’s method, adapted to the use of Atlas.ti [35]. The process included the following steps: (1) reading all interview notes to acquire a general sense of the content; (2) identifying significant statements related to the phenomenon; (3) formulating meanings from these statements; (4) organizing formulated meanings into clusters of themes and subthemes; (5) developing an exhaustive description of the phenomenon; and (6) returning to participants for member checking to validate the findings and interpretations.

The researchers gathered original and unpublished information. Participation was requested voluntarily from the nurses, who signed an informed consent form. The criteria, strategies, and activities implemented during the interviews were carefully designed to ensure the credibility, transferability, dependability, and reflexivity of the study, following qualitative research standards [44]. These methodological considerations are detailed in Table 2, which outlines the interview technique, the interview guide as the primary instrument, and the scheduling flexibility provided to participants based on their availability.

A total of nine key informants were included in the study. Each participant was assigned a unique identification code that reflects their order of participation, gender, and age. For example, the code P1M45 corresponds to Participant 1, male, aged 45. This coding system ensured the confidentiality and organization of the collected data. The demographic characteristics and identifiers of the participants are presented in Table 3, allowing for a clearer understanding of the sample profile used in the research.

The interview guide, designed by the primary researcher, was evaluated by five experts and refined based on their feedback. It included open-ended questions addressing institutional policies, professional identity, competencies, and experiences in delivering humanized care. No repeat interviews were conducted. All interviews were carried out via WhatsApp video calls and lasted approximately 30 min. Only the participant and interviewer were present during each session.

In accordance with participants’ requests, interviews were not audio-recorded. Instead, detailed field notes were taken during and immediately after each interview. The interviewer also used a reflective diary to enhance self-awareness and ensure methodological rigor.

Interviews were conducted until theoretical saturation was reached. After data analysis, researchers returned key findings to participants for member checking, validating the interpretations drawn from their narratives.

Two researchers independently coded the data using Atlas.ti 22 software. Themes were not pre-established; rather, they emerged inductively from participant narratives following Colaizzi’s seven-step method. Codes were organized into four thematic categories and multiple subthemes, forming a coding tree that guided analysis and discussion. The audit trail and codebook were maintained to ensure transparency and traceability.

### 2.5. Data Analysis

Data analysis was conducted following Colaizzi’s descriptive phenomenological method [35]. Initially, the researchers engaged in bracketing by explicitly setting aside their preconceived notions about humanized care. Subsequently, immersion in the data was achieved through multiple readings of detailed field notes, allowing deep familiarity with participants’ narratives. Significant statements directly related to humanized care were extracted, from which meanings encapsulating the essence of each statement were formulated. These formulated meanings were then clustered into thematic categories. Finally, these themes were integrated into an exhaustive description of the participants’ lived experiences, providing comprehensive insight into the essence of humanized care. The findings were validated through member checking to ensure accurate representation of participants’ experiences.

Although audio recordings were not available due to participants’ preferences, data analysis was carried out using rigorously detailed field notes. These notes, taken during and immediately after each interview, included verbatim responses, key phrases, tone of voice, emotional cues, and contextual impressions. After each session, the interviewer expanded and clarified the notes to preserve fidelity to participants’ expressions. Two researchers independently reviewed these enriched field notes and conducted line-by-line coding using Atlas.ti 22. Themes were not pre-established; instead, they were derived inductively following Colaizzi’s seven-step method. Clusters of meanings were discussed among the research team, and discrepancies were resolved through consensus. Although verbatim quotes are not included due to the lack of recordings, the trustworthiness of the findings was ensured through member checking, triangulation, peer debriefing, and audit trail documentation.

### 2.6. Ethical Considerations

This study was conducted in accordance with the ethical principles outlined in the Declaration of Helsinki. Ethical approval was obtained from the Arzobispo Loayza National Hospital (Peru), on 3 June 2024. All participants received detailed information about the objectives, procedures, risks, and benefits of the study. They were informed of their right to withdraw at any time without consequences. Written informed consent was obtained from each participant before conducting the interviews

## 3. Results

Although direct quotations are not included due to the nature of data collection and participants’ preference for non-recorded interviews, the analysis was grounded in detailed field notes and systematically coded responses. Each insight was attributed to participants using identification codes (for example, P2F25 indicating Participant 2, Female, 25 years old), reflecting their order of participation, gender, and age. This approach ensured consistency and transparency in representing the views that supported the emergence of key themes and subthemes throughout the results.

The findings obtained from the interviewees’ testimonies were processed, allowing for their categorization, sub-categorization, coding, and systematization, yielding the following findings: one overarching category, “Experience in the construction of humanized care,” and its four interconnected subcategories, identified from the expectations of the nursing professionals. This structure reveals the holistic understanding these professionals have of humanized care, encompassing not only direct actions but also the broader institutional and professional context. The subsequent table elaborates on the emergent themes and key ideas within these categories, providing a detailed map of the elements crucial for fostering a humanizing approach in healthcare settings, as seen in Table 4.

To build humanizing healthcare environments, nursing professionals provide humanized care, as shown by the testimonies collected. Despite global efforts to strengthen healthcare systems, institutional health policies continue to fall short in preventing dehumanization. Contributing factors include high patient volumes, excessive medical and administrative workloads, prolonged work shifts, and insufficient resources. These issues result in dissatisfaction and a negative perception of service quality [47]. Although institutional policies promote humanized care, their effectiveness is hindered by limited human resources, medical staff, and outdated infrastructure, which restrict mobility for patients and families and worsen navigation due to poor signage in hospitals.

## 4. Discussion

Concerning professional image and identity, these elements are key to patients’ perceptions of quality care. Watson emphasizes that nursing is an art that involves deeply understanding the patient’s emotions as if they were one’s own [47,48]. A nurse (P1M36) noted that their professional image is often distorted by taking on tasks meant for other professionals, leaving behind their core responsibilities of patient-centered care. Individualized attention not only addresses patient needs but also enhances their emotional well-being and dignity. Nurses (P2F25, P3F30) also stressed the importance of developing psychosocial competencies and decision-making abilities. Others (P7F31, P8F37, P9F56) highlighted that effective communication and emotional intelligence are vital in offering comfort in times of suffering and uncertainty.

On the topic of strengths and challenges of humanized care, it was stated that this approach is not just a philosophy but a way of life. Nurses must be trained to face all kinds of hospital situations. Prioritizing patient-centered care and promoting continuous improvement strategies is crucial [20,47]. One nurse (P1M36) emphasized that autonomy and cultural sensitivity are foundational, while others (P7F31, P8F37, P9F56) reinforced the idea that competencies strengthen professional roles. According to testimonies (P2F25, P3F30, P4F47, P5F30, P6F45), active participation in public policy and updated academic curricula foster leadership, public health impact, and social transformation. Ethical values are essential to build strong interpersonal relationships [49].

Regarding competencies for humanized care, the testimonies highlight the importance of both technical and relational skills. Professional identity is built through daily practice, ongoing education, and reflective learning [50]. This promotes environments where patients feel heard and valued. Humanized care not only improves patient experiences but also enhances job satisfaction, creating a positive cycle in healthcare delivery [51]. Continuous training and institutional support are necessary to ensure care that respects patients’ dignity.

Despite technological advances, nurses continue to play an irreplaceable role in providing emotional support to patients and their families. Testimonies (P1M36, P2F25, P3F30, P4F47) stressed that holistic, humanized, and quality care must be monitored and evaluated over time. Nurses (P6F45, P7F31, P8F37, P9F56) also noted that although there is willingness to provide this care, increasing demands and limited medical and human resources lead to complaints and dissatisfaction. The biomedical model further hinders humanized practices [52]. Institutional policies must be rethought to prioritize patient-centered approaches [53]. Despite existing policies, practical implementation is fragile due to poor coordination with administrative staff and other healthcare professionals.

Essential competencies for humanized care include communication skills that promote empathetic dialog [54], active listening, ethical and cultural training [55], and critical self-reflection [56]. Nurses (P1M36, P2F25, P3F30, P4F47) emphasized that ongoing education is necessary to stay up to date and improve patient outcomes. Collaboration with other health professionals allows for comprehensive care. Others (P5F30, P6F45, P7F31, P8F37, P9F56) shared that interdisciplinary teamwork and empathy improve patient recovery and quality of life. These elements are foundational for transforming healthcare delivery and ensuring that patients feel truly cared for.

While this study is limited to the Peruvian context, the findings resonate with broader international research on humanizing nursing care. In particular, the concept of “caring contact”—encompassing intentional touch, eye contact, and attentive presence—emerges as a key component of relational care [50,56]. Although not explicitly addressed as “caring massage” in the interviews, participants’ descriptions of therapeutic communication and emotional support align with this theoretical framework. The integration of such approaches in clinical training may bridge the gap between local practice and global standards in nursing care. Future research should explore how these international models can be adapted to local healthcare environments to foster cross-cultural relevance.

## 5. Implications of the Findings

The findings of this phenomenological study highlight the profound meanings that nursing professionals attribute to the experience of providing humanized care. Rather than being a procedural framework, humanized care is revealed as an emotional, relational, and ethical commitment embedded in the nurse-patient encounter. This insight shifts the focus from structural process optimization to the subjective and affective dimensions of care, requiring institutions to reorient their strategies.

The lived experiences shared by participants indicate that humanized care cannot thrive under fragmented systems where emotional labor is undervalued, workloads are excessive, and administrative demands overpower relational presence. A central implication is the need for organizational reforms that center emotional and interpersonal dynamics as key indicators of quality (not just efficiency or compliance). Hospital policies should incorporate relational competencies into their performance metrics and job descriptions, emphasizing the value of empathy, cultural sensitivity, and emotional intelligence in everyday practice.

Another critical implication concerns nursing education and professional development. Curricula must go beyond technical training to include structured modules on phenomenological reflection, ethical care, narrative medicine, and the role of emotions in clinical encounters. Incorporating reflective practice early in nurses’ formation would help normalize emotional expression, self-awareness, and ethical discernment, which are vital to delivering person-centered care.

Additionally, the findings imply a redefinition of leadership in healthcare environments. Nurse managers and hospital administrators must be trained to recognize humanized care not as a soft skill, but as a pillar of institutional integrity and patient satisfaction. This calls for leadership models that support emotional well-being, encourage compassionate teamwork, and reduce professional burnout (thereby creating environments where nurses feel psychologically safe to engage authentically with patients).

Participants’ narratives also reveal that interpersonal connection is often impeded by environmental and systemic limitations—such as inadequate space, insufficient time, or lack of privacy. These insights suggest that designing more “relationally intelligent” physical spaces, allocating protected time for emotional engagement, and removing barriers to nurse-patient interaction could have measurable impacts on care quality.

Moreover, these lived experiences help reframe the role of nurses as system-sensemakers. Nurses are not merely implementers of care protocols but interpreters of suffering and advocates for meaning-making within the healthcare process. Their unique positioning allows them to detect dissonances between institutional values and actual practices, making them vital contributors to ethical redesign and compassionate innovation in healthcare delivery.

Finally, the findings emphasize the importance of longitudinal support and recognition. Humanized care is not a one-time act but a continuous stance, requiring reinforcement through institutional mechanisms such as reflective supervision, peer support programs, and awards or incentives for excellence in compassionate care. Recognizing and valuing these efforts could elevate humanized care from being a moral ideal to becoming a standardized expectation within healthcare systems.

## 6. Recommendations

To cultivate a healthcare system truly centered on human dignity, several interconnected reforms are essential. Firstly, institutional policy must undergo a significant revision, moving beyond theoretical frameworks of humanized care to concrete practices supported by adequate resources. This necessitates ensuring sufficient staffing levels, appropriate infrastructure, and comprehensive training programs. Practical considerations such as clear signage, facilitating patient mobility, and streamlining administrative processes to reduce the burden on both staff and patients deserve specific attention.

Secondly, strengthening the professional identity of nursing professionals is paramount. Continuous support is needed to ensure their roles are not diminished by tasks falling outside their expertise. This involves actively promoting the inherent value of personalized care and reinforcing crucial emotional and psychosocial competencies from the very beginning of their academic journey.

Furthermore, healthcare institutions should prioritize the development of emotional intelligence and communication skills among nurses. Implementing structured programs focused on these areas will equip them to effectively navigate sensitive situations involving pain, grief, and uncertainty, enabling them to offer meaningful emotional support to both patients and their families.

The foundation for a humanized healthcare workforce lies in evolving and updating academic curricula. Educational institutions should integrate modules that explicitly address leadership skills, cultural sensitivity, ethical considerations, and the specific competencies required for humanized care. This proactive approach will ensure that future generations of nurses are well-prepared to meet the diverse and complex needs of their patients.

To ensure the sustained delivery of high-quality humanized care, establishing robust monitoring systems is crucial. This includes implementing effective feedback mechanisms, tracking relevant quality indicators, and conducting regular evaluations of training programs to facilitate continuous improvement over time.

Recognizing that comprehensive care requires a unified effort, healthcare institutions should actively encourage interdisciplinary collaboration. Fostering strong working relationships among nurses, doctors, social workers, therapists, and other healthcare professionals will ensure a holistic and empathetic approach to patient care. This teamwork not only enriches the patient experience but also contributes to the professional growth and development of all involved.

Finally, both institutional and public recognition of humanized care efforts is vital. Acknowledging and celebrating the dedication of nursing professionals who consistently demonstrate excellence in providing compassionate care can significantly boost staff morale and enhance public trust in the healthcare system. This recognition should extend to include meaningful career incentives and ensure that humanized care is a visible priority within public health agendas. To further cultivate a culture of continuous improvement, training programs must incorporate dedicated spaces for reflective practice and critical thinking, allowing nurses to analyze their care delivery, learn from their experiences, and refine their approach to providing truly holistic patient care.

## 7. Limitations

While the qualitative phenomenological approach allowed for an in-depth understanding of participants’ lived experiences, several limitations should be acknowledged. First, the study was conducted with a small, purposively selected sample of nine nursing professionals from a single public hospital in Peru. Although this sample size is appropriate for phenomenological inquiry, which prioritizes depth over breadth, it may limit the generalizability of findings to other contexts or healthcare systems.

Second, due to the preferences of the participants, interviews were not audio-recorded. Instead, detailed field notes were taken during and immediately after each interview. While the researchers made every effort to ensure accurate and comprehensive transcription (including validation of notes and member checking) there remains the possibility that some nuances of expression or tone were lost without recorded data. This limitation may have affected the richness and interpretive depth of some testimonies.

Third, the study is geographically limited to the Peruvian context. Although it provides valuable insights into the experiences of nurses operating within this specific healthcare system, the cultural, institutional, and systemic conditions in Peru may differ significantly from those in other countries. As such, caution should be exercised when attempting to extrapolate the findings to international settings.

Nonetheless, many of the themes identified (particularly those relating to emotional support, therapeutic communication, and the importance of institutional policy) resonate with broader international research on humanizing nursing care. The concept of “caring contact,” for example, which includes intentional touch, eye contact, and presence, aligns with the theoretical framework described by global literature. While this study did not explicitly explore caring massage or similar interventions, participant narratives reflected practices consistent with these approaches. This suggests potential avenues for further research on how such frameworks can be integrated into both local training programs and broader cross-cultural nursing education.

Future studies should consider expanding the participant pool to include multiple institutions and regions, incorporating audio or video recording to enhance data accuracy, and exploring comparative perspectives to strengthen the international relevance of findings.

## 8. Conclusions

Drawing from the multifaceted insights gathered, this study underscores the critical and evolving understanding of humanized care within the nursing profession. The findings reveal that the construction of a truly humanizing healthcare environment is not a singular endeavor but rather a complex interplay of institutional support, professional identity, relational dynamics, individual competencies, and deeply held values.

The experiences shared by nursing professionals highlight the foundational role of supportive institutional policies that translate theoretical ideals into tangible resources and practices. This includes adequate staffing, infrastructure, and ongoing training that empowers nurses to deliver compassionate care without undue burden. Furthermore, a strong sense of professional identity, nurtured through continuous support and recognition, is essential for nurses to confidently embrace their holistic role and advocate for patient-centered approaches.

The study also illuminates the crucial significance of emotional intelligence and effective communication as cornerstones of the nurse-patient relationship. The ability to connect with patients on an emotional and spiritual level, to actively listen and respond with empathy, directly elevates the quality of care and fosters trust. However, the path to fully humanized care is not without its obstacles. The identified limitations, such as resource scarcity, resistance to change, and the emotional toll on both professionals and patients, necessitate proactive strategies to mitigate these challenges.

Ultimately, the cultivation of humanized care hinges on a workforce equipped with comprehensive competencies that extend beyond technical skills to encompass attitudinal, emotional, digital, and ethical dimensions. Grounded in core values such as empathy, respect, integrity, and solidarity, these competencies enable nurses to provide care that is not only effective but also deeply person-centered and culturally sensitive.

Finally, achieving widespread and sustainable humanized care requires a systemic and multi-pronged approach. It demands a commitment from healthcare institutions to prioritize policies and resources that support this model of care. It necessitates the continuous development and empowerment of nursing professionals, fostering their skills, values, and professional identity. And it calls for an ongoing dialog and reflective practice within the profession to adapt and evolve in response to the ever-changing needs of patients and the healthcare landscape. By embracing these interconnected elements, the healthcare system can move towards a future where compassionate, ethical, and truly human-centered care is not an aspiration, but a consistent reality.

## Figures and Tables

**Table 1:** Interview guide for health professionals.

Item Number	Question Asked
Item 1	From your experience, how can a humanizing healthcare environment be built?
Item 2	Do you believe institutional health policies promote humanized care?
Item 3	What professional profile should a caregiver providing humanized care have?
Item 4	What are the strengths and limitations that affect humanized care?
Item 5	What competencies should a professional have to provide humanized care?

**Table 2:** Quality criteria established in the research.

Criteria	Strategies	Activities Carried Out
Credibility	Interaction with Participants	Nursing professionals were invited voluntarily and provided informed consent before participating. All interviews were conducted via WhatsApp video calls in private settings chosen by the participants to ensure comfort and confidentiality.
	Observation	The primary researcher used a pre-designed field note template during the WhatsApp video interviews to document non-verbal expressions (e.g., facial tone, emotional pauses), tone of voice, and contextual impressions. These notes supported the interpretive depth of the analysis.
	Member Checking	Key themes and interpretations were summarized and shared with participants via follow-up WhatsApp messages. Participants reviewed and confirmed the accuracy of the researchers’ interpretations, allowing for clarification and validation of meaning.
	Triangulation of Theories	The analysis was theoretically triangulated using Jean Watson’s Theory of Human Caring and Virginia Waldow’s humanized care model [45,46]. Watson emphasizes ethical, emotional, and transpersonal relationships in care, while Waldow focuses on humanized care as a culturally rooted, holistic practice. Together, these frameworks grounded the interpretation of participant narratives.
Transferability	Thorough Description	Participant profiles (such as age, gender, role, years of experience) and institutional context were described in detail to allow readers to assess the potential applicability of findings to similar healthcare settings.
Dependability	Audit Trail	The research process—including participant selection, interview scheduling, field note documentation, coding, and peer debriefing—was recorded in a structured log. Data were managed and stored using Mendeley and Atlas.ti to ensure consistency and traceability.
Confirmability	Peer Review and Data Management	Two researchers independently reviewed the field notes and coding outputs. Regular team discussions were held to resolve differences, ensuring that findings were based on participant data rather than researcher bias.
Reflexivity	Reflexive Journal	The lead researcher maintained a reflexive journal throughout the study to record personal assumptions, reactions, and methodological decisions. This journal was revisited regularly to reduce bias and enhance analytic transparency.

**Table 3:** Sample characteristics.

Sample	Current Position	Gender	Age	Code
S1	Clinical Nurse—Teacher	Male	36 years old	P1M36
S2	Head of the Nursing Department	Female	25 years old	P2F25
S3	Nursing Supervisor	Female	30 years old	P3F30
S4	Clinical Nurse	Female	47 years old	P4F47
S5	Nursing Teacher	Female	30 years old	P5F30
S6	Clinical Research Nurse	Female	45 years old	P6F45
S7	Head of the Medicine Department	Female	31 years old	P7F31
S8	Head of the Emergency Department	Male	37 years old	P8F37
S9	Director of Nursing	Female	56 years old	P9F56

**Table 4:** Thematic analysis of humanized care experiences.

Category	Subcategory	Emerging Topic	Key Ideas
Experience in the Construction of Humanized Care	Institutional health policies	Humanizing actions in professional practice	Evaluation and feedback on compliance with humanized care policies Patient-centered approach Continuous training and +education Creation of a humanizing environment Patient and community involvement Recognition and certification of the institution
	Professional image and identity	Profile of the nurse with a holistic and humanizing approach	Vocation of service Patient-centered care management (pcc) Research, innovation-creativity-art Health education Application of artificial intelligence Patient-family empowerment and participation in care Leads, autonomous and participatory Interdisciplinary and multidisciplinary work Emotional and spiritual connection Address cultural care respecting diversity Ability to resolve conflict situations
	Strengths and Challenges of Humanized Care	Actions that elevate the nurse-patient relationship	Comprehensive and holistic care approach Effective therapeutic communication Empowerment and participation of the patient and family User and professional satisfaction
		Limiting Actions That Hinder Humanized Care	Lack of human resources and medical supplies Resistance to change Emotional stress experienced by professionals and users High patient expectations regarding healthcare services
	Competencies for Humanized Care	Comprehensive Professional Competencies	Technical and scientific competencies Attitudinal competencies Digital competencies Emotional and communicative competencies Educator role Clinical management and primary care Researcher role
		Cultivating Values	Empathy and compassion Respect Integrity and autonomy Solidarity Responsibility Equity and social inclusion Active listening Cooperation, integration, and collaboration High ethical sense and responsibility
		Skills	Ability for active listening Teamwork capacity Soft skills Social and group skills Communication skills Use of digital tools Problem-solving: Capacity to educate in health

## Data Availability

The raw data supporting the conclusions of this article will be made available by the authors on request.
